# T173. GABA AND GLUTAMATE IN PATIENTS WITH 22Q11.2 DELETION SYNDROME AND HEALTHY VOLUNTEERS AND THE RELATION WITH COGNITION: A RANDOMIZED DOUBLE-BLIND 7TESLA PHARMACOLOGICAL MRS STUDY

**DOI:** 10.1093/schbul/sby016.449

**Published:** 2018-04-01

**Authors:** Claudia Vingerhoets, Desmond Tse, Mathilde van Oudenaren, Esther van Duin, Dennis Hernaus, Jan Ramaekers, Jaap Janssen, Grainne McAlonan, Oswald Bloemen, Therese van Amelsvoort

**Affiliations:** 1 Maastricht University; 2 Academic Medical Center; 3 Institute of Psychiatry; 4 GGz Centraal/Maastricht University

## Abstract

**Background:**

22q11.2 deletion syndrome (22q11DS) is characterized by a microdeletion on the long arm of chromosome 22. The clinical phenotype of this syndrome is highly variable but symptoms include cognitive impairment, heart malformations, auto-immune problems and a high risk of developing a psychotic disorder. One of the genes located in the deleted region is PRODH which encodes proline dehydrogenase (PRODH). This enzyme is involved in converting proline to glutamate (GLU). GLU is involved in the pathophysiology of psychosis, particularly in cognitive symptoms (Lewis and Moghaddam 2006). Gamma-aminobutyric acid (GABA) is involved in cognition and psychosis as well (Vinkers et al. 2010). With this study we aimed to investigate GLUergic and GABAergic reactivity in the anterior cingulate cortex (ACC) and striatum in medication-free patients with 22q11DS with no psychiatric history and healthy controls (HC).

**Methods:**

This was a randomized double-blind placebo controlled cross-over study. Groups were matched for age and gender. 12 patients with 22q11DS (mean age 35 years) and 20 HCs (mean age 31 years) were enrolled in the study. GABA and GLU, levels in the ACC and striatum were obtained twice with 7Tesla Magnetic Resonance Spectroscopy (MRS, STEAM): once after placebo and once after oral administration of 50 mg. riluzole (agent with anti-glutamate and pro-GABA action). Striatal and ACC GLU/GABA ratios were computed as well as GLUergic and GABAergic reactivity (placebo minus riluzole). In addition, within the 22q11DS group, the relationship between cognitive functions (memory and attention) measured with the CANTAB and GABA, GLU, GLU/GABA ratio, GABAergic reactivity and GLUergic in the ACC and striatum were examined.

**Results:**

Analyses of Covariance (ANCOVA) showed no baseline group differences in glutamate and GABA levels and GLU/GABA ratios (corrected for fraction of cerebral spinal fluid, CSF) in both brain regions. A repeated measures ANCOVA showed a trend level significant increase in striatal GABA concentrations after (p= 0.065). Riluzole had no significant effect on GLU (p= 0.303) and GLU/GABA ratios (p= 0.150) in the striatum. No medication X group interaction effects were found. Riluzole had no significant effect on GABA (p= 0.101), GLU (p= 0.847) and GLU/GABA ratio (p= 0.108) in the ACC. No group main effects and no medication X group interactions effects were found. However, a significant negative correlation was found between verbal memory (r= -0.650, p= 0.030) and ACC GLU levels, as well as GLUergic reactivity (r= -0.733, p= 0.010). Moreover, in the 22q11DS group, a significant negative correlation was found between attention (target sequence detection) and ACC GLU levels (r= -0.704, p= 0.016) as well as GLU/GABA ratio (r= -0.602, p= 0.050). Furthermore, sustained attention was positively associated with ACC GABA levels (r= 0.700, p= 0.024) and negatively associated with GLU/GABA ratio r= -0.639, p= 0.047) in these patients. Finally, a positive correlation was found between visual memory and striatal GLU levels (r= 0.616, p= 0.043).

**Discussion:**

The present study did not demonstrate differences in ACC and striatal GLU and GABA levels, nor in GLUergig or GABAergic reactivity in response to riluzole between 22q11DS patients and controls. However, these results suggest a role for GLU and GABA in cognition in the 22q11DS group. Therefore, influencing these neurotransmitter systems might enhance cognitive functioning in these patients. More studies are required to replicate these findings.

